# Classification of microbial *α*-amylases for food manufacturing using proteinase digestion

**DOI:** 10.1002/fsn3.133

**Published:** 2014-06-11

**Authors:** Takumi Akiyama, Takeshi Yamazaki, Atsuko Tada, Yusai Ito, Noriko Otsuki, Hiroshi Akiyama

**Affiliations:** 1 National Institute of Health SciencesTokyo, Japan; 2 Jissen Women's UniversityTokyo, Japan; 3 Kyoritsu Women's UniversityTokyo, Japan

**Keywords:** Endoproteinase Lys-C, HPLC, peptide, trypsin, *α*-amylase

## Abstract

Enzymes produced by microorganisms and plants are used as food additives to aid the processing of foods. Identification of the origin of these enzyme products is important for their proper use. Proteinase digestion of *α*-amylase products, followed by high performance liquid chromatography (HPLC) analysis, was applied to *α*-amylase from the mold *Aspergillus* species, the bacteria *Bacillus* species, and the actinomycetes *Saccharomonospora* species. Eighteen commercial products of *α*-amylase were digested with trypsin and endoproteinase Lys-C and HPLC analyzed. For some proteinase/sample combinations, the area of the intact *α*-amylase peak decreased and new peaks were detected after digestion. The presence and retention times of the novel peaks were used to group the products. The results from this method, called the proteinase digestion–HPLC method, allowed the classification of the *α*-amylase products into 10 groups, whereas the results from sodium dodecyl sulfate polyacrylamide gel electrophoresis allowed their classification into seven groups.

## Introduction

Enzymes produced by microorganisms and plants are used worldwide as food additives to aid the processing of manufactured foods such as bread, wine, vinegar, and cheese. For example, *α*-amylase is generally used in the production of starch sugars and breads, and lipase is generally used to produce oils and fats or to improve the quality of oils. The enzymes approved as food additives for use in Japan are shown by law in the List of Existing Food Additives (Notification No. 120 [16 April 1996], Ministry of Health and Welfare, Japan). In this list, the enzymes are classified based on their function, such as *α*-amylase, lipase, cellulase, or protease. Therefore, the classified enzymes contain enzyme products derived from various sources.

The amino acid sequences of enzymes derived from different sources are varied, and the enzyme properties, such as optimal catalytic activity assay conditions, are diverse. Therefore, the properties of enzyme products, that is, optimal pH and substrate specificities, may differ among species and even among strains within a species. This is particularly true for enzymes from microorganisms. The identification of the origin of an enzyme product is very important for its appropriate application in food manufacturing, and also from the standpoint of safety; it is important to exclude enzyme products from strains that are not approved for use in food. Generally, food additives including food enzyme must comply with specifications which include information to adequately identify the food additive including origin and to describe the acceptable criteria of purity. The specifications are developed by manufacturer corporations, industrial association, international regulatory organizations, or national regulatory authorities. Specifications developed by producer corporations often precede those by other organizations. Improvement of specifications of a product can increase reliability of the product for customer companies. Therefore, it would be beneficial if methods for identifying the origins of enzyme products were included in these specifications.

The differences in the properties of enzyme products, that is, optimal pH and substrate specificity, are assessed by enzyme activity assays. However, identifying the origin of an enzyme product using enzyme activity assays is difficult since the test samples must be stored properly before their assay to maintain optimal catalytic activity. Moreover, some enzyme activity assays require specific substrates and are time consuming. A simple chemical analysis of the enzyme proteins in an enzyme product as a test for identifying the origin of the enzyme product would be useful.

We have established a simple method for classifying enzyme products using sodium dodecyl sulfate polyacrylamide gel electrophoresis (SDS-PAGE) (Akiyama et al. 2010). SDS-PAGE analyses provide useful data for approximately 100 food enzyme products, although there are some limitations for *α*-amylase (1,4-*α*-d-glucan glucanohydrolase; EC 3.2.1.1), which hydrolyzes the *α*-glucose bonds of polysaccharides such as starch. Many *α*-amylase products from microorganisms have been developed for industrial use. We obtained 18 products extracted from microorganisms such as *Aspergillus* species, *Bacillus* bacteria, and the actinomycetes *Saccharomonospora* species. Each product from *Aspergillus niger*, *A. oryzae*, and three species of *Bacillus* provided one main band. The origin of each product was identified based on the mobility of the main bands. However, it was difficult to identify the origin of products from *Bacillus* without using visibility or the mobility of the weak bands (separation patterns D–F in Table 1).

**Table 1:** List of *α*-amylase products and their species origin (provided by the manufacturer).

Genus	Species	Sample number
*Aspergillus*	*Aspergillus foetidus*^1^	1
	*Aspergillus niger*	2
	*Aspergillus oryzae*	3, 4, 5, 6, 7
	*Aspergillus niger* and *A. oryzae*	8
*Bacillus*	*Bacillus amyloliquefaciens*	9
	*Bacillus licheniformis*	10, 11, 12, 13
	*Bacillus subtilis*	14, 15, 16, 17
*Saccharomonospora*	*Saccharomonospora viridis*^2^	18

1 Former Latin name = *Aspergillus aureus*.

2 Former Latin name = *Thermomonospora viridis*.

In this study, we used proteinase digestion followed by high performance liquid chromatography (HPLC) analysis to identify the origins of *α*-amylase products. Trypsin and endoproteinase Lys-C were used because they are commercially available and their properties are extensively studied. Trypsin is a serine protease that cleaves peptide chains at the carboxyl side of the amino acids lysine or arginine. Endoproteinase Lys-C cleaves proteins at the carboxyl side of lysine residues. The combination of the results from this study and SDS-PAGE analyses enabled more precise classification of *α*-amylase products than classification using SDS-PAGE results alone.

## Material and Methods

### *α*-Amylase products

*α*-Amylase products commercially available in Japan were kindly provided by the Japan Food Additives Association (JFAA) together with information about their origin, provided by the manufacturers. Eighteen products from eight sources were obtained (Table 1). All these food enzyme products are produced by microorganisms. The microbial sources are identified by their Latin names. Former names that were used in the List of Existing Food Additives in Japan (Notice No. 56 [23 May 1996], Division of Food Chemistry, Environmental Health Bureau, Ministry of Health and Welfare, Japan) are shown in Table 1 and the producer microorganisms in Table 2.

**Table 2:** Digestion-based grouping of *α*-amylase products.

			SDS-PAGE		Trypsin		Lys-C	
Information on the origin^1^	Combined grouping	Sample number^2^	Separation pattern	Molecular weights of major proteins (kDa)	Separation pattern	Retention time of major peaks (min)	Separation pattern	Retention time of major peaks (min)
*Aspergillus foetidus*	I	1	A	87, 50	NO^3^		l	9.7, 10.0, 10.4, 11.7, 12.9, 13.2, 15.0, 15.3
*Aspergillus niger*	II	2	B	64	h	5.2, 5.6, 6.2, 6.4, 6.7, 7.7, 7.8, 8.0, 9.2, 10.1, 11.0, 11.3, 11.7, 12.3, 12.6, 12.8, 13.0, 13.6, 14.7, 14.9, 15.4, 16.8	m	7.9, 10.6, 13.3, 13.9, 15.3
*Aspergillus oryzae*	III	3, 4, 5, 7	C	50	NO		n	8.2, 10.0, 11.4
	IV	6	C	50	NO		l	9.7, 10.0, 10.4, 11.7, 12.9, 13.2, 15.0, 15.3
*Aspergillus niger* and *A. oryzae*	I	8	A	87, 50	NO		l	9.7, 10.0, 10.4, 11.7, 12.9, 13.2, 15.0, 15.3
*Bacillus amyloliquefaciens*	V	9	D	54, [24]^4^	i	12.3, 16.9	NO	
*Bacillus licheniformis*	VI	10, 11, 13	E	54, [43]^4^	j	13.7, 16.8	NO	
	VII	12	E	54, [43]^4^	k	14.2, 17.1	o	14.2, 17.2
*Bacillus subtilis*	VIII	14, 15, 17	F	54	i	12.3, 16.9	NO	
	IX	16	E	54, [43]^4^	j	13.7, 16.8	p	13.7, 16.9
*Saccharomonospora viridis*	X	18	G	(32), (23)^5^	NO		NO	

1 The origin was provided by the manufacturer.

2 Samples with the same information on the origin and group are placed in the same row.

3 No apparent digestion was observed.

4 Figures in brackets indicate weak bands.

5 Figures in parentheses indicate bands that appear only when a large amount of sample is loaded.

### Proteinases and reagents

Trypsin from bovine pancreas and endoproteinase Lys-C from *Lysobacter enzymogenes* were sequence grade products and were purchased from Roche Diagnostics (Basel, Switzerland).

### Proteolysis of *α*-amylase samples

Optimal conditions were chosen for each proteinase. For trypsin digestion, 0.1 mol/L Tris-HCl pH 8.5, 0.2 mg of target protein, and 10 *μ*g of trypsin were contained in a 50-*μ*L reaction mixture. For Lys-C digestion, 40 mmol/L Tris-HCl pH 8.5, 1 mmol/L EDTA, 10 *μ*g of target protein, and 0.5 *μ*g of Lys-C were contained in a 50-*μ*L reaction mixture. The amount of *α*-amylase sample was adjusted so that each reaction mixture had the same concentration of total protein. The reaction mixtures were incubated at 37°C for 4 h.

### HPLC

Each reaction mixture was diluted fourfold with Milli Q water, then 10 *μ*L of the diluted mixture was separated using HPLC. The HPLC system was an ACQUITY UPLC system (Waters, Milford, MA). An ACQUITY UPLC BEH C18 column (2.1 mm i.d. × 100 mm, Waters) and a VanGuard Pre-column (2.1 mm i.d. × 5 mm, Waters) were used at 30°C. The mobile phases A and B were 0.1% trifluoroacetic acid and acetonitrile, respectively. A linear gradient from 5% B to 75% B in 30 min was applied. The flow rate was 0.4 mL/min. The wavelength of the detector was 210–400 nm.

Chromatograms of a digested sample and a control without any proteinase were compared for every product. When specific peaks were found for a product, the product were compared with other products exhibiting specific peaks and grouped.

## Results and Discussion

### Products from Aspergillus sp

**Figure 1:**
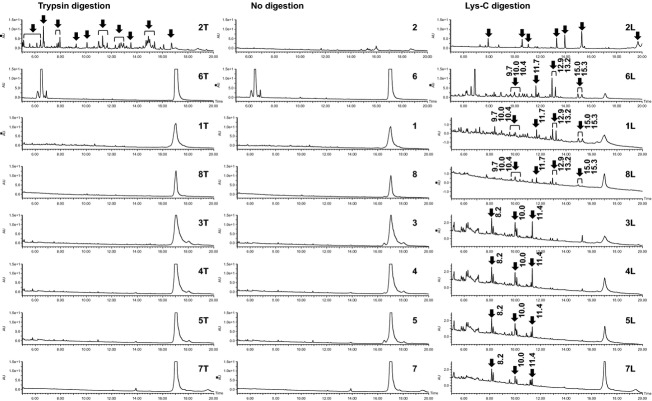
HPLC chromatograms of *Aspergillus* products. T, trypsin digestion; L, Lys-C digestion. The retention times of the peaks are shown. Peaks diminished following digestion are marked with open arrows. New peaks and peaks intensified after digestion are marked with closed arrows.

To identify the origin of enzyme products derived from *Aspergillus* sp., we analyzed the digested product samples using HPLC. Figure 1 shows chromatograms of products 1–8, which are derived from *Aspergillus* sp.

**Figure 2:**
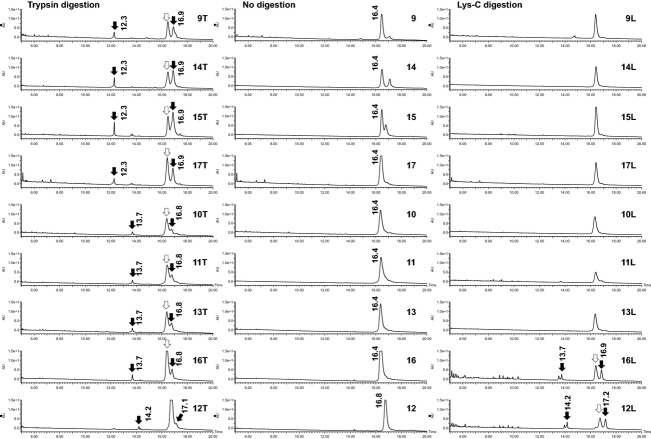
HPLC chromatograms of *Bacillus* products. T, trypsin digestion; L, Lys-C digestion. The retention times of the peaks are shown. Peaks diminished following digestion are marked with open arrows. New peaks and peaks intensified after digestion are marked with closed arrows.

Chromatograms of trypsin-digested samples were compared to those of undigested samples. As shown in Figure 1, several peaks are observed after digestion of product 2 from *A. niger* (Fig. 1-2, 2T). No differences between digested and undigested samples were found for other products derived from *Aspergillus* sp. (Fig. 1-1T, 3T, 4T, 5T, 6T, 7T, 8T, Table 1).

Chromatograms of Lys-C-digested samples were compared with those of undigested samples. As shown in Figure 1, several peaks are observed in product 2 from *A. niger*. The chromatographic profiles of trypsin-digested and Lys-C-digested samples are completely different; the separation patterns were termed “h” and “m,” respectively (Table 2). In the chromatograms of *Aspergillus* products 1 and 3–8, the main peak around 17 min appears to be intact *α*-amylase protein because the peak also is detected in the undigested samples (Fig. 1-1, 3, 4, 5, 6, 7, 8). In contrast, the area of the main peak is lower and new peaks are detected after Lys-C digestion (Fig. 1-1L, 3L, 4L, 5L, 6L, 7L, 8L). These chromatograms show two separation patterns (Table 2). The chromatograms of products 1, 6, and 8 have peaks at 9.7, 10.0, 10.4, 11.7, 12.9, 13.2, 15.0, and 15.3 min (Fig. 1-1L, 6L, 8L) and give separation pattern l. Products 3, 4, 5, and 7 give separation pattern n, in which peaks are detected at 8.2, 10.0, and 11.4 min (Fig. 1-3L, 4L, 5L, 7L).

Several *Aspergillus α*-amylases have been cloned and sequenced. For example, two kinds of *α*-amylases have been cloned from *A. oryzae* and are registered as AMYA1_ASPOR and AMYA3_ASPOR in the SWISS-PROT database. Both are 478 amino acid long polypeptides with 20 lysine residues. If one of these polypeptides is cut at the carboxyl side of all the lysine residues by Lys-C, 21 polypeptide chains with molecular weights ranging from 0.5 to 6.0 kDa would be produced. In our experiments, several peaks were observed after Lys-C digestion of products 3, 4, 5, 6, and 7. These peaks are therefore probably peptides with lysine at the carboxyl end.

Product 8 is derived from both *A. niger* and *A. oryzae*, according to the manufacturer's information. Its chromatographic profile is similar to that of product 6, which is derived from *A. oryzae*. However, the peaks found in the chromatograms of digested product 2, which is derived from *A. niger*, were not observed in the chromatograms of digested product 8.

### Products from Bacillus sp

To identify the origin of the enzyme products derived from *Bacillus* sp., we analyzed the digested product samples using HPLC. Figure 2 shows the chromatograms of products 9–17, which are derived from *Bacillus* sp.

In all chromatograms, the main peak around 16 min appears to be intact *α*-amylase protein because the peaks are detected in the undigested samples (Fig. 2-9, 10, 11, 12, 13, 14, 15, 16, 17). As shown in Figure 2-9T, 10T, 11T, 12T, 13T, 14T, 15T, 16T, 17T, for most products, the area of the main peak in the undigested sample is lower after trypsin digestion. Two new peaks (one with a shorter retention time, around 12–14 min, and one at 17 min) in addition to the peak of intact *α*-amylase protein are detected for all products after digestion. The chromatograms show three separation patterns based on the retention times of these peaks (Table 2). For product 9, 14, 15, or 17, the first peak is detected at 12.2 min (Fig. 2-9T, 14T, 15T, 17T). The separation pattern of these products was termed “i.” Products 10, 11, 13, and 16 give separation pattern j, since the first peak for these products is detected at 13.7 min (Fig. 2-10T, 11T, 13T, 16T). For product 12, which gives separation pattern k, the first peak is detected at 14.2 min (Fig. 2-12T).

As shown in Figure 2-12T and 16T, after Lys-C digestion, the area of the main peaks of products 12 and 16 are smaller and other two peaks are detected. The separation patterns for products 12 and 16 were termed “o” and “p,” respectively, because the retention times of the peaks around 14 and 17 min are slightly different. No difference between Lys-C-digested and -undigested samples is found for other products derived from *Bacillus* sp. (Fig. 2-9L, 10L, 11L, 13L, 14L, 15L, 17L).

*α*-Amylases from *B. amyloliquefaciens*, *B. licheniformis*, and *B. subtilis* have been cloned. They are registered in the SWISS-PROT database as AMY_BACAM, AMY_BACLI, AMYM_BACLI, and AMY_BACSU. Ghasemi et al. (2007) reported the generation and identification of two large polypeptides after trypsin digestion and SDS-PAGE analysis of the *α*-amylase from *B. licheniformis*. N-terminal sequencing of the peptides after purification by SDS-PAGE revealed that trypsin cleaved the Arg127–Val128 peptide bond. The *α*-amylase used in that study contains 20 Arg and 28 Lys residues. The authors speculated that Arg127 resides in a flexible region and was subject to trypsin attack. In our study, trypsin digestion of *α*-amylases derived from *B. licheniformis* (products 10, 11, 12, and 13) gave two peaks in HPLC analysis. This result suggests that the peaks detected by HPLC analysis are the N-terminal and C-terminal peptides produced by peptide bond cleavage on the carboxyl side of Arg127.

### Products from *Saccharomonospora viridis*

For product 18, which is produced from *Saccharomonospora virides*, the digested and undigested samples give identical chromatograms with and without trypsin or Lys-C digestion (data not shown). Complete genomic sequence of a type strain of *S. viridis* provided a probable *α*-amylase gene, which is registered in the TrEMBL database as C7MZU7_SACVD (Pati et al. 2009). Calculated molecular weight of the predicted protein of this entry is 58 kDa. It is not known that this entry represents active *α*-amylase proteins of product 18. Absence of any bands that correspond to this predicted protein in the SDS-PAGE analysis of product 18 (Akiyama et al. 2010) might indicate that amount of the *α*-amylase protein is not enough to be detected in HPLC analysis in this study (Table 2).

### Grouping of *α*-amylase products

*α*-Amylase products were grouped based on the results of analyses using the SDS-PAGE method developed in our previous report and the proteinase digestion–HPLC method presented in the present paper (Table 2).

From SDS-PAGE analysis, *Aspergillus* products 1–8 were divided into groups A (products 1 and 8), B (product 2), and C (products 3–7). Products in group C were derived from *A. oryzae*. Digestion of product 6 with Lys-C provided a chromatogram that was clearly different from those of the other four products (products 3, 4, 5, and 7) and similar to those of products 1 and 8. Therefore, *Aspergillus* products were divided into groups l (products 1, 6, and 8), m (product 2), and n (products 3, 4, 5, and 7) based on results from the proteinase digestion–HPLC method. The results from trypsin digestion divided products 1–8 into group h (product 2) and a group that gave no novel peaks (products 1 and 3–8). The results from trypsin digestion were not as informative as the results from Lys-C digestion. The combination of the SDS-PAGE method and the proteinase digestion–HPLC method allowed products 1–8 to be divided into groups I (products 1 and 8), II (product 2), III (products 3, 4, 5, and 7), and IV (product 6).

When *Bacillus* products 9–17 were separated with SDS-PAGE, the main band of each product had similar mobility. It was difficult to distinguish products from *Bacillus* without using presence/absence or the mobility of the weak bands. The results of trypsin digestion allowed the products to be grouped into groups i (products 9, 14, 15, and 17), j (products 10, 11, 13, and 16), and k (product 12) based on the retention times of specific peaks observed in the HPLC chromatograms. Group j was further divided into subgroups o (products 10, 11, and 13) and p (product 16) because Lys-C digestion of product 16 provided new peaks (separation pattern p), whereas no digestion of the other products was apparent. Products 9–17 were divided into groups V (product 9), VI (products 10, 11 and 13), VII (product 12), VIII (products 14, 15 and 17), and IX (product 16) using the results from both the SDS-PAGE and proteinase digestion–HPLC methods.

### Information obtained through the proteinase digestion–HPLC method

To our knowledge, there are no reports on the proteinase digestion of *Aspergillus α*-amylases. The information obtained by the proteinase digestion–HPLC method allowed more precise grouping of *α*-amylases than their grouping using results from the SDS-PAGE method.

Products 3–7 from *A. oryzae* were divided into two groups, III and IV (Table 2). The *α*-amylases from several strains of *A. oryzae* are commercially available as a processing aid and are used in food manufacturing. Groups III and IV might represent *α*-amylases from two of these strains. Product 2 from *A. niger*, which provides a different pattern from other *Aspergillus* products upon SDS-PAGE analysis (Akiyama et al. 2010), gave specific results in this study. The proteinase digestion–HPLC method can identify this product among eight products tested in this study. Product 1 from *A. foetidus* and product 8 from *A. niger* and *A. oryzae* belong to the same group by the above grouping criteria. *Aspergillus foetidus* and *A. niger* are classified to the same subgenus, *Circumdati*, under genus *Aspergillus* (Klich 2002). The resemblance between the *α*-amylases of these two species might reflect their taxonomical proximity.

In SDS-PAGE analysis, products from *Bacillus* gave intense bands with similar mobility (Akiyama et al. 2010). The extensive sequence homology between cloned *α*-amylases from *B. amyloliquefaciens*, *B. licheniformis*, and *B. subtilis* can explain this result. It was reported that *B. amyloliquefaciens*, *B. licheniformis*, and *B. subtilis* belong to the same cluster when sequences of 16S rRNA of 69 *Bacillus* species were compared (Goto et al. 2000). These findings suggest that the proteinase digestion–HPLC method can help group some *Bacillus* products. Products 10–13 from *B. licheniformis* were divided into two groups, VI and VII (Table 2). The proposal HPLC method clarified the difference between group VIII and IX in a manner not possible using the SDS-PAGE method.

## Conclusions

The *α*-amylases from *Aspergillus* were classified into four groups based on results from the SDS-PAGE and proteinase digestion–HPLC methods. The results from SDS-PAGE and proteinase digestion–HPLC analyses allowed the *α*-amylases from *Bacillus* to be divided into five groups. All 18 products, which were classified into seven groups by the SDS-PAGE method, were reclassified into 10 groups. To the best of our knowledge, this is the first report to use analyses of enzyme proteins to distinguish the origin of food enzyme products for industrial use.
